# Polycyclic Aromatic Hydrocarbons Concentrations in Drinking Water in Villages along the Huai River in China and Their Association with High Cancer Incidence in Local Population

**DOI:** 10.1155/2015/762832

**Published:** 2015-11-24

**Authors:** En chun Pan, Hong Sun, Qiu jin Xu, Qin Zhang, Lin fei Liu, Xiao dong Chen, Yan Xu

**Affiliations:** ^1^Huai'an Center for Disease Control and Prevention, Huai'an, Jiangsu 223001, China; ^2^Jiangsu Provincial Center for Disease Control and Prevention, Jiangsu Road 172, Nanjing 210009, China; ^3^Chinese Research Academy of Environmental Science, Beijing 100012, China

## Abstract

This study aims to evaluate the carcinogenic risk of PAHs in the drinking water of counties along the Huai River in China and study their associations with high cancer incidence in local population. We investigated 20 villages with high cancer incidence rates as the risk group and 20 villages with low rates as the control group. Water samples from each village were collected in the winter and summer seasons to analyze the concentrations of 16 PAHs. The carcinogenic risks of the PAHs were calculated for each village using a health risk assessment approach. Results showed that PAHs concentrations in 27.2% of the water samples were higher than the allowable values in China. However, no significant difference in water PAHs concentrations was observed between the risk and control groups (*P* > 0.05), and no correlation was found between water PAHs concentrations and cancer incidence in these villages. The average upper bound carcinogenic risks were less than 1 × 10^−4^ in both groups. In conclusion, PAHs were present in the drinking water of the studied villages, but their carcinogenic risks remained within acceptable limits. PAHs in local drinking water might not be the major environmental cause of the high cancer incidences.

## 1. Introduction

The incidence of cancer among rural residents along the Huai River in China is higher than national average level, and scientists and the Chinese public have attributed this high cancer incidence to water pollution [1, 2]. Pollution of drinking water by PAHs is an important public health issue that has attracted great concern [3]. Previous studies have already detected polycyclic aromatic hydrocarbons (PAHs) in the water of the Huai River [4]. PAHs are known carcinogens, and their presence in drinking water may be associated with the high local cancer incidence observed; however, no health risk assessment of PAHs in drinking water has been performed before. Previous epidemiological studies show that PAHs exposure in occupational workers is associated with many kinds of cancers [5–7], and PAHs are listed as priority pollutants by the United States Environmental Protection Agency (US EPA) and the European Union. Nowadays in Chinese villages, rapid economic development and urbanization continue to consume large amounts of fossil fuel and biomass each year and thus produce more PAHs, which could enter surface water through atmospheric fallout. So human health risk assessment (HHRA) for PAHs in local drinking water has been strongly encouraged.

Typically, HHRA involves four steps: data collection and analysis, exposure assessment, toxicity assessment, and risk characterization. After monitoring data for the target contaminant is gathered, the path, frequency, duration, and magnitude of actual human exposure to the contaminant can be estimated. The types of adverse health effects associated with contaminant exposure and the relationships between exposure dose and adverse effects are then identified. The final risk characterization summarizes and combines the outputs of the exposure and toxicity analyses to assess the contaminant's quantitative and qualitative risks [8, 9]. Probabilistic approaches such as a Monte Carlo simulation provide flexible tools for estimating the uncertainties and stochastic properties of contaminant exposure and toxicity. Consequently, probabilistic HHRAs have been successfully used to assess the potential adverse health effects of water contaminants [10–12].

Jiangsu province has the second highest cancer mortality rate in China. Between 2004 and 2005, the malignant tumor mortality rate in Jiangsu province was 151.97 per 100,000 people, much higher than the national average of 123.72 per 100,000 people. The incidence of cancer, particularly from stomach and esophageal cancers, in Huai'an city is significantly higher than the average level in Jiangsu province. This study is conducted to evaluate the association of PAHs in drinking water in three counties with high cancer incidence rates in Huai'an city. We combine epidemiological investigations and HHRA to establish an evaluation model that can be used for the investigations of environmental carcinogens.

## 2. Materials and Methods

### 2.1. Research Design and Ethics

We used an ecological study design and performed the special investigation. A total of 40 villages were investigated in this study based on incidence rates of malignant tumor during 2008 to 2010. The incidence and mortality data of tumor were recorded using International Classification of Diseases (ICD) system, and the monitored tumors included all types of malignant tumors (ICD10: C00.0-C97). Given the small population in these studied villages (1214–5590 persons in a village) and because the tumor register information was available from 2008, the three-year average of cancer incidence and mortality rates from 2008 to 2010 were statistically analyzed. And we used 3-year average incidence rates of malignant tumors to assign villages to the risk or control group.

**Figure 1:**
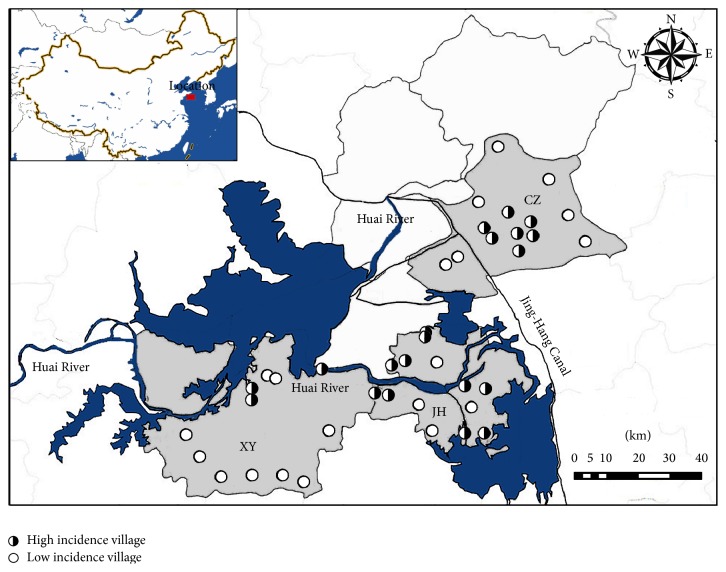
Location of the research area and sampling points.

We selected 20 villages with low cancer incidence rates as the control group and 20 villages with high cancer incidence rates as the risk group. Control group villages were selected from the top 10 towns with the lowest cancer incidence rates in the three counties, while villages in the risk group were selected from the top 10 towns with the highest cancer incidence. In each selected town with high cancer incidence rate, the three-year average of incidence rates of each cancer studied was calculated for each village; the top two villages with the highest rates were assigned to the risk group. Similarly, the top two villages with the lowest rates in the selected town with low cancer rate were assigned to the control group. The investigated villages were shown in Figure 1, which were selected from the same municipal district with similar life customs, and no known factors associated with cancer, such as hepatic B virus or* Helicobacter pylori* infections, among the resident populations were recorded between the risk and control groups. In each village, samples of surface water (from rivers or ponds), shallow groundwater (from shallow wells), and deep groundwater (from taps) were collected during both summer and winter seasons.

This study was conducted according to the guidelines laid down in the Declaration of Helsinki, and all procedures involving human subjects/patients were approved by the Health Bureau of Huai'an Municipal and the Ethics Committee of the Huai'an Municipal CDC. Written informed consent was obtained from all subjects.

### 2.2. Sampling and PAHs Analysis

Samples were collected in 1000 mL amber glass bottles with Teflon lined tops. The water samples were collected from the 40 villages in both August 2010 (summer season) and February 2011 (winter season). For each village, 3 water samples were collected for each season, respectively. For surface water and shallow groundwater, 10 L of water was collected in 10 different places and mixed together to measure. And 1 L of tap water was sampled at each village. Shallow groundwater samples were collected 10–15 m below the ground's surface, and the water pumped during the first 5 min of sampling was discarded. Tap water in the investigated areas was obtained from deep groundwater (at least 100 m deep). All water samples were transported to the laboratory and kept at 4°C in sealed containers prior to PAHs analysis.

Water samples were analyzed at the Beijing Institute of Petrochemical Technology. Standard solutions of 16 PAHs (10 mg/L in acetonitrile) were purchased from Supelco (Supelco Inc., PA, USA). The PAHs analyzed included naphthalene (Nap), acenaphthylene (Acy), acenaphthene (Ace), fluorene (Fl), phenanthrene (Phe), anthracene (Ant), fluoranthene (Flu), pyrene (Pyr), benzo[a]anthracene (BaA), chrysene (Chy), benzo[a]pyrene (BaP), benzo[b]fluoranthene (BbF), benzo[k]fluoranthene (BkF), dibenzo[a,h]anthracene (DahA), indeno[1,2,3-cd]pyrene (IcdP), and benzo[g,h,i]perylene (BghiP). The C18 extraction cartridges used in the study were purchased from Chromaband (Manchery-Nagel, Germany), and a solid-phase extraction (SPE) vacuum manifold was used to concentrate and purify solvent extracts. Analytical-reagent grade cyclohexane, acetone, biphenyl, and methanol were also used in this study (Merck, Germany).

Water samples were extracted using SPE system according to the established procedures [13]. Each sample was eluted three times in the HLB tube in 10 mL of dichloromethane and methanol (9 : 1), followed by three elutions in the Envi-C18 tube with 10 mL of hexane and methanol (7 : 3). The resulting extract was dried under a gentle stream of nitrogen at 40°C and then transferred into a 500 mL microvial. Phe-D10 (0.1 mg/L) was added as the internal standard, and the vial was refrigerated until analysis.

PAHs extracts were analyzed using an HP-5 column (30 m × 0.25 mm × 0.25 *μ*m) on an Agilent 7890 gas chromatograph (GC) coupled to an Agilent 5975 mass spectrometer (MS). GC/MS was performed using EPA method 8270D as previously described [13]. Briefly, the GC/MS was set to an initial column temperature of 70°C with an initial holding time of 2 min and then subjected to a 5°C/min increase in temperature to 290°C for 4 min. The injector and detector temperatures were 250 and 300°C, respectively. Helium was used as the carrier gas, and a flow rate of 2 mL/min was employed. PAHs concentrations were identified based on retention time and confirmed by comparison of the mass spectra obtained with a reference library. Calibration curves were plotted using seven concentrations of standard prepared PAHs solutions ranging from 2 ng/L to 2000 ng/L.

PAHs in the water blanks were not detected or much lower than the detection limits of the method (1–5 ng/L in this study). Deuterated internal standards were used to compensate for losses involved in the sample extraction and clean-up. The internal standards in water were determined with good precision. The recoveries were 70.4 ± 6.7% for Nap-d8, 72.1 ± 8.7% for Ace-d10, 95.1 ± 10.7% for Phe-d10, 87.5 ± 9.8% for Chr-d12, and 85.4 ± 7.8% for Per-d12 in water samples, respectively.

### 2.3. Health Risk Assessment

#### 2.3.1. Exposure Assessment

The concentrations of multicomponent PAHs were converted into their BaP equivalents (BaPeq) for exposure assessment. BaPeq concentrations were calculated by multiplying the concentrations of carcinogenic PAHs with their corresponding BaP-relative potency equivalency factors (PEFs) for seven PAHs given by the US EPA in 1993, including BaA, Chy, BaP, BbF, BkF, DahA, and IcdP [17].

Two possible paths of water exposure were considered: ingestion and dermal absorption. Exposure doses of ingestion and dermal absorption were calculated using (1) and (2), respectively; these equations were adopted from the US EPA [18]:

(1) $$\begin{array}{c} CDIi=\frac{Cw\times IR\times EF\times ED}{BW\times AT}, \end{array}$$

where CDIi is the chronic daily BaPeq intake via ingestion (mg/kg·day), Cw is the BaPeq concentration in water (mg/L), IR is the ingestion rate of water (L/day), EF is the exposure frequency (350 days/year in this study), ED is the exposure duration (years), BW is the body weight (kg), and AT is the average time (days; 25,550 days in this study). One has

(2) $$\begin{array}{c} CDId=\frac{Cw\times SA\times Kp\times ET\times EF\times ED\times CF}{BW\times AT}, \end{array}$$

where CDId is the chronic daily BaPeq intake by dermal absorption (mg/kg/day), SA is the exposed dermal surface area (cm^2^), Kp is the dermal BaP permeability coefficient (cm/h), ET is the amount of exposure per day (h/day), and CF is the transformation factor (1 L/1000 cm^3^).

Exposure parameters and their probability distributions are shown in Table 1. BW, SA, IR, and ET were calculated according to statistical data from China [15]. Given the limited statistical data available, other parameters, such as Kp and SF, were directly derived from reference values from the US EPA [14].

#### 2.3.2. Toxicity and Risk Characterizations

Cancer slope factor (SF) quantitatively defines the relationship between the exposure dosage of a carcinogen and its corresponding cancer risk. According to the Integrated Risk Information System of the US EPA [16], the geometric mean (GM) of the SF of BaP is 7.3 (mg/kg/day)^−1^; this value was used as the SF during risk assessment in this study. However, as this SF value is expressed as oral administrative dose derived from rodent feeding studies whereas dermal exposure is presented as absorbed dose, the SF value for dermal exposure was adjusted with the gastrointestinal absorption adjustment factor (AAF) [18]. Furthermore, the point estimate of the gastrointestinal absorption of BaP is 92% in the dose-response studies from which the cancer SF for BaP was derived [19]. As such, the SF for dermal BaP exposure is equal to 7.3 (mg/kg/day)^−1^/92% = 7.9 (mg/kg/day)^−1^.

Carcinogenic risks (CRs) of ingestion and dermal exposure were calculated using (3), which was adapted from the US EPA [14]:

(3) $$\begin{array}{c} CR=CDI\times SF, \end{array}$$

where CR is the probability of developing cancer over a lifetime as a result of exposure to a contaminant, CDI is CDIi or CDId, and SF is the corresponding slope factor. The total carcinogenic risk of BaP in water was calculated as the sum of the CRs from ingestion and dermal exposure.

#### 2.3.3. Uncertainty Analysis

Monte Carlo simulation (*n* = 10, 000) was used to quantify uncertainties and their impact on risk estimation. Values and sources of input parameters were obtained from the* Exposure Factors Handbook* of US EPA [14] and China EPA [15] and were listed in Table 1. Sensitivity analyses were also performed to identify the significance of input parameters and calculated rank correlation coefficients between the input and output values of Monte Carlo simulations. Monte Carlo simulation and sensitivity analyses were performed using Oracle Crystal Ball software version 11.2.

### 2.4. Statistical Analyses

The normal distribution of PAHs concentrations achieved by* BoxCox* transformation depends on a number of best-fitted lambda values [20]. The normal distribution of malignant tumor incidence in the villages was verified by the Anderson-Darling and Kolmogorov-Smirnov tests. Comparisons of PAHs concentrations between different seasons and water types were performed by ANOVA coupled with a nonparametric equivalent, such as the Kruskal-Wallis (three-group comparisons) or Wilcoxon rank sum (two-group comparisons) test. SPSS 18.0 for Windows (SPSS Inc., Chicago, IL, USA) was used to analyze statistical correlations and perform ANOVA. A *P* value <0.05 was considered statistically significant.

## 3. Results and Discussion

### 3.1. Cancer Incidence and Mortality Rates in the Control and Risk Groups

The average crude incidence of cancer in the risk group was 397.47 per 100,000 people with the lowest incidence being 235.22 per 100,000 people (Table 2). By contrast, the average cancer incidence of the control group was 134.89 per 100,000 people with the highest incidence being 189.83 per 100,000 people. Cancer mortality rates significantly correlated with incidence rates in the studied villages (*r* = 0.78, *P* < 0.01). Our classifications for groups were referenced from the Chinese National Central Cancer Registry, which reports an age-standardized malignant tumor incidence of 146.87 per 100,000 for the Chinese population in 2009 [21].

### 3.2. Distribution Characteristics of PAHs

A total of 232 water samples from 40 villages were obtained and analyzed. PAHs concentrations detected in the winter and summer seasons were shown in Table 3. DahA was not detected in any sample, and BaP could not be detected in 9.86% of the samples. The maximum BaP concentration found among the water samples was 158.06 ng/L and the maximum BaPeq concentration observed was 159.01 ng/L. These PAHs concentrations are consistent with other studies performed in China [8, 22]. PAHs levels in uncontaminated groundwater are usually in the range of 0–5 ng/L. Concentrations above this level indicate contamination by PAHs mainly through industrial point sources and shipyards, atmospheric deposition, and urban runoff [23]. Our reported total PAHs concentration was much higher than previous report for the Mississippi River, USA (GM = 115 ng/L) [24], but was similar with the report for the Almendares River in Cuba (GM = 2784 ng/L) [25].

According to the Chinese safety standard for drinking water, the BaP concentration must be less than 10 ng/L [26]; in this study, abnormal BaP concentrations were found in 25.9% (60/232) of the samples. When BaPeq was considered instead of BaP, the corresponding rate was 42.2% (98/232). When the total PAHs level was compared with the Chinese national limit (2000 ng/L), PAHs in 27.2% (63/232) of all samples exceeded the limit. We noted, however, that the allowable BaP concentrations in drinking water in the USA and Egypt are 200 ng/L [27] and 700 ng/L [28], respectively. Differences between allowable BaP concentrations in drinking water in China and other countries necessitate performance of risk assessment for the rest of this study.

The concentrations of most PAHs in the summer season were significantly higher than their corresponding concentrations in the winter season (Table 3). This result suggested that precipitation might introduce PAHs to drinking water sources, or the higher levels of dissolved organic carbon were present in surface water in the summer season. This temporal distribution of PAHs in water was consistent with another study that reported much lower PAHs in winter than in spring or summer [29]. Total PAHs concentrations were significantly higher in surface water than in groundwater regardless of seasons (*P* < 0.01 in both seasons, Table 3). However, carcinogenic PAHs concentrations in surface water were significantly higher than those in groundwater only in the summer season (*P* = 0.33 in winter season, and *P* < 0.01 in summer season, Table 3).

### 3.3. PAHs Concentrations between the Risk and Control Group

As shown in Table 4, the risk group showed the concentration of BaPeq similar to those in the control group; no statistically significant difference was found between the risk and control groups on BaPeq (*P* = 0.55). The distribution of carcinogenic PAHs also showed no significant difference between groups (*P* = 0.85 for all samples in each group, Table 4). The only significant difference between two groups was observed in terms of Acy concentrations in surface water (*P* = 0.04, Table 4): higher Acy levels were observed in the control group instead of the risk group.

We further compared PAHs distributions in different water sources between groups by seasons. The results in Table 5 indicated that total PAHs, carcinogenic PAHs, and BaPeq concentrations showed no significant difference between the risk and control groups. In the summer season, BkF levels in deep groundwater were higher in the risk group than in the control group (*P* = 0.03, Table 5).

### 3.4. Health Risk Assessment

#### 3.4.1. Exposure Assessment and Health Risk Characterization of PAHs

Daily BaPeq exposure doses through ingestion and dermal adsorption as well as their carcinogenic risks were calculated via both deterministic and probabilistic approaches. Here, BaPeq concentration was used as Cw in (1) and (2); other parameters used in our deterministic approach are shown in Table 1. As the US EPA considers a cancer risk range of 1 × 10^−6^ to 1 × 10^−4^ an acceptable risk management range [14], this range was used as the criterion in our study. We found that 87.3% of all samples show a risk > 1 × 10^−6^ but none of them show a risk > 1 × 10^−4^; these results indicate that the carcinogenic risks of PAHs in water from the villages under study are within the acceptable risk range. Carcinogenic risks for villagers who used groundwater were significantly lower than for those who used surface water (*P* = 0.03). Although most of the carcinogenic risk values in the risk group were higher than those in the control group (Table 6), no statistically significant difference was found between these groups (*P* = 0.44).

The Cw generated from the best-fit distribution of the detected BaPeq concentrations was used in our probability-based approach (Monte Carlo simulation). In general, the mean, 5th percentile, median, and 95th percentile calculated risk using Monte Carlo approach were higher than but consistent with values calculated using the deterministic approach (Table 6).

#### 3.4.2. Sensitivity Analysis

**Figure 2:**
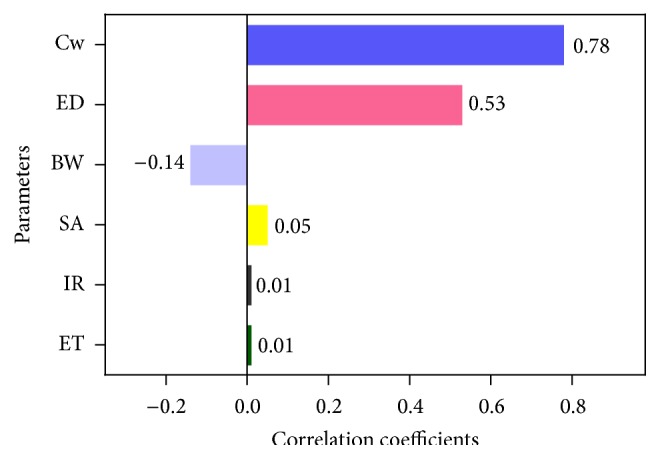
Sensitivity analysis for carcinogenic risk assessment of PAHs in drinking water.

Quantitative sensitivity analysis was conducted to identify parameters with the most influence on output carcinogenic risk values (Figure 2). These parameters are presented as rank order correlation coefficients. BaPeq concentration in water (Cw), with a correlation coefficient of 0.78, was found to be the most influential variable, followed by ED, with a correlation coefficient of 0.53. While BW negatively influenced carcinogenic risk, SA, IR, and ET exerted the least influence; these findings may be a result of the lack of variation in these parameters. The SA variation in this assessment was limited; however, because the SA was replaced with body surface area (assuming bathing every day), this assessment may be expected to produce an overestimated risk. These sensitivity analysis results are consistent with a previous report that also included Kp and SF in its model [8]. The probability distributions of Cw and ED must be determined more thoroughly to improve the accuracy of our results.

#### 3.4.3. Uncertainty Analysis

We identified factors (Cw and ED) with the most influence on final carcinogenic risk using Monte Carlo simulations. If these parameters exhibited broader distributions (i.e., if their SD/mean increased), these parameters would more strongly affect the final risk calculations. This study is limited by the Cw value of BaPeq, which was measured only once during the winter and summer seasons; such infrequent sampling may yield a BaP concentration that is not representative of entire seasons. Therefore, continued monitoring of BaP concentrations is necessary.

We overestimated ED (exposure duration) for each type of water source because we calculated an ED of 70 years, which is much higher than the real value. In all of the villages studied, residents drank deep groundwater (tap water) for 20 years; both surface and shallow well water were used as drinking sources in the past. Supposing that PAHs in groundwater were increased with economic developments, the Cw of PAHs in groundwater was also overestimated; therefore, the actual exposure risks for the elders were much lower than our calculated values. We further estimated possible maximal exposure risks using the Cw in the winter and summer seasons as independent values for personal exposure. This condition also results in overestimation of our results. For personal exposure, the average BaPeq during the winter and summer seasons is a better estimate of actual exposure.

SF and Kp were derived from animal experiment results, and uncertainties may be contributed to this study by the conversion of test results from animals to humans. The point estimate of SF for BaP suggested by the US EPA is derived from oral administration and affected by the absorption efficiency in different treatments, which may require AAF adjustment. The AAF was estimated as 92% from a critical animal study and may also contribute uncertainties to this study. Given that Kp values are unique to specific PAHs, the use of the Kp for BaP as a representative value for all other carcinogenic PAHs presents another source of uncertainty. The probability distribution of population parameters such as BW and SA was obtained from statistical data of Chinese adults, and then such parameters may overestimate the exposure risk for elderly people. Considering these uncertainties, however, the present study used parameters of BW, IR, ET, and SA specific for eastern Chinese rural populations as obtained from a recent exposure factors handbook of the Chinese population [15]; use of these parameters ensures relatively specific estimations in this study.

### 3.5. Limitation and Policy Implication

Incidence of cancer involved many complicated factors and needed long-term exposure. The values of HHRA in this study were based on the present exposure data, which might not correlate well with the current incidence of cancer in these villages. However, by evaluating and comparing the carcinogenic risk of PAHs exposed from drinking water between risk group and control group, we finally showed the safety of PAHs in current drinking water.

## 4. Conclusion

PAHs concentrations were higher in summer season than in winter season. Surface water contained relatively high concentrations of PAHs and was not suitable for drinking in the studied villages. PAHs concentrations in drinking water were higher than national limits in the investigated villages but showed no significant difference between risk and control groups. We estimated that the average carcinogenic risk of PAHs in water was less than 1 × 10^−4^ in both groups. Thus, supposing the PAHs in water kept stable or increasing in the past years, we could conclude that the PAHs in drinking water were not the major risk factor for high cancer incidence in these villages.

## Figures and Tables

**Table 1:** Values and probability distributions of parameters used in exposure assessment.

Definition	Units	Distributions	Lifetime exposure	Reference
Average time (AT)	Day		25550	[14]
Body weight (BW)	Kg	Lognormal	62.1	[15]
Dermal permeability coefficient (Kp)	cm/h		1.2	[14]
Exposure duration (ED)	Year	Uniform	0–70	[14]
Exposure frequency (EF)	Day/year		350	[14]
Exposure (ET)	h/day	Lognormal	0.15	[15]
Ingestion rate (IR)	L/day	Lognormal	2.39	[15]
Surface area (SA)	cm^2^	Lognormal	16000	[15]
Slope factor (SF)	(mg/kg/day)^−1^		7.3	[16]

**Table 2:** Incidence and mortality rates of cancer in the investigated villages (per 100,000 people).

Group	*N*	Variables	Mean	SD^a^	Min	Max	Median	P95
Control	20	Incidence	134.89	50.33	71.56	189.83	116.74	189.83
		Mortality	140.06	71.73	29.82	216.20	136.05	216.20

Risk	20	Incidence	397.47	92.35	235.22	576.84	388.31	548.89
		Mortality	313.44	73.83	198.33	407.38	332.19	404.41

^a^Standard deviation.

**Table 3:** Distribution and comparison of PAHs by season.

	Winter season GM (95% CI) (ng/L)				Summer season GM (95% CI) (ng/L)				*P* (winter versus summer)			
	Surface water (SW)	Shallow groundwater (SG)	Deep groundwater (DG)	*P* ^a^	Surface water (SW)	Shallow groundwater (SG)	Deep groundwater (DG)	*P* ^a^	*P* (SW)	*P* (SG)	*P* (DG)	*P* (all)
Nap	104.9 (86.4, 127.3)	41.5 (34.7, 49.6)	40.8 (33.5, 49.8)	0.00^*∗∗*^	2054 (1127.1, 3743.2)	757.6 (375.7, 1527.6)	739.1 (430.8, 1267.9)	0.05	0.00^*∗∗*^	0.00^*∗∗*^	0.00^*∗∗*^	0.00^*∗∗*^
Acy	10.7 (8.1, 14)	4.9 (4, 6)	5.7 (4.7, 6.9)	0.00^*∗∗*^	32.7 (19.2, 55.8)	18.2 (11.6, 28.6)	12.5 (7.4, 21.1)	0.02^*∗*^	0.00^*∗∗*^	0.00^*∗∗*^	0.03^*∗*^	0.00^*∗∗*^
Ace	5.9 (4.4, 7.7)	2.1 (1.6, 2.7)	2.9 (2.2, 3.7)	0.00^*∗∗*^	96.1 (63.3, 145.9)	45.8 (32.2, 65.3)	36.0 (25.3, 51.3)	0.00^*∗∗*^	0.00^*∗∗*^	0.00^*∗∗*^	0.00^*∗∗*^	0.00^*∗∗*^
Fl	39.2 (30.6, 50.1)	14.5 (12, 17.6)	16.6 (13.4, 20.5)	0.00^*∗∗*^	421.5 (247.8, 717.1)	200.9 (129, 312.9)	167.7 (102.4, 274.7)	0.02^*∗*^	0.00^*∗∗*^	0.00^*∗∗*^	0.00^*∗∗*^	0.00^*∗∗*^
Phe	45.3 (35.1, 58.6)	19.6 (13.8, 28)	24.0 (18.2, 31.8)	0.00^*∗∗*^	730.5 (447.9, 1191.2)	339.1 (204, 563.7)	316.9 (201, 499.7)	0.02^*∗*^	0.00^*∗∗*^	0.00^*∗∗*^	0.00^*∗∗*^	0.00^*∗∗*^
Ant	4.4 (3.4, 5.8)	2.6 (2, 3.4)	2.6 (1.9, 3.5)	0.01^*∗*^	46.9 (28.3, 77.8)	29.9 (18, 49.7)	30.5 (20, 46.5)	0.39	0.00^*∗∗*^	0.00^*∗∗*^	0.00^*∗∗*^	0.00^*∗∗*^
Flu	18.9 (15.8, 22.7)	16 (10.8, 23.7)	10.0 (6.3, 15.8)	0.06	102.3 (71.5, 146.4)	68.8 (45.9, 103.1)	50.6 (36.8, 69.6)	0.02^*∗*^	0.00^*∗∗*^	0.00^*∗∗*^	0.00^*∗∗*^	0.00^*∗∗*^
Pyr	18.0 (14, 23)	17.5 (12.1, 25.4)	13.3 (10.1, 17.4)	0.27	66.4 (47.1, 93.6)	55.2 (37.3, 81.6)	41.9 (31.1, 56.3)	0.18	0.00^*∗∗*^	0.00^*∗∗*^	0.00^*∗∗*^	0.00^*∗∗*^
BaA	1.8 (1.4, 2.4)	1.9 (1.1, 3.2)	1.8 (1.2, 2.6)	0.98	9.4 (7.2, 12.3)	8.9 (6.7, 11.8)	5.8 (4.5, 7.4)	0.02^*∗*^	0.00^*∗∗*^	0.00^*∗∗*^	0.00^*∗∗*^	0.00^*∗∗*^
Chy	8.3 (6.9, 9.9)	6.1 (4.1, 9)	5.3 (3.8, 7.6)	0.21	31.3 (22.4, 43.9)	19.6 (13.1, 29.5)	15 (12.6, 17.8)	0.00^*∗∗*^	0.00^*∗∗*^	0.00^*∗∗*^	0.00^*∗∗*^	0.00^*∗∗*^
BbF	4.9 (2.8, 8.5)	3.7 (2.1, 6.6)	3.4 (1.8, 6.2)	0.65	2.9 (2, 4.2)	2.6 (1.7, 3.8)	1.9 (1.5, 2.4)	0.22	0.13	0.26	0.05	0.01^*∗*^
BkF	7.5 (2.1, 26.7)	4.3 (2.1, 8.8)	5.1 (2.1, 12.4)	0.50	43.9 (31.5, 61.3)	22.4 (14.5, 34.6)	29.8 (22.6, 39.2)	0.03^*∗*^	0.00^*∗∗*^	0.00^*∗∗*^	0.00^*∗∗*^	0.00^*∗∗*^
BaP	6.0 (3.6, 10.2)	5.3 (2.5, 11.1)	3.0 (1.9, 4.6)	0.14	11.7 (9, 15.4)	7.7 (5.4, 10.8)	6.0 (4.8, 7.7)	0.00^*∗∗*^	0.01^*∗*^	0.30	0.00^*∗∗*^	0.00^*∗∗*^
IcdP	6.2 (2.9, 13.2)	8.2 (2, 33.4)	3.6 (1.2, 11.1)	0.53	9.9 (5.7, 17)	7.1 (4.9, 10.4)	5.8 (4.2, 8)	0.06	0.27	0.78	0.33	0.24
BghiP	5.4 (2.5, 11.7)	3.0 (1.5, 5.9)	2.7 (1.3, 5.7)	0.28	11.7 (8.4, 16.3)	7.1 (5.4, 9.3)	6.0 (4.9, 7.4)	0.00^*∗∗*^	0.03^*∗*^	0.00^*∗∗*^	0.00^*∗∗*^	0.00^*∗∗*^
∑PAHs	296.9 (253.5, 347.6)	157.1 (122, 202.4)	148.8 (121.6, 182)	0.00^*∗∗*^	4054.7 (2427.2, 6773.6)	1877 (1190.4, 2959.5)	1611.7 (1035.7, 2508.1)	0.01^*∗*^	0.00^*∗∗*^	0.00^*∗∗*^	0.00^*∗∗*^	0.00^*∗∗*^
∑(PAHs-c)^b^	28.6 (19.5, 41.9)	18.2 (11.2, 29.6)	16.6 (11, 25.1)	0.33	122.7 (92.8, 162.2)	83 (62.6, 110)	69.6 (59, 82.2)	0.00^*∗∗*^	0.00^*∗∗*^	0.00^*∗∗*^	0.00^*∗∗*^	0.00^*∗∗*^
BaPeq	4.4 (2.2, 9)	2.1 (0.9, 4.9)	2.9 (1.6, 5.1)	0.64	10.7 (8, 14.4)	8.1 (6.6, 10)	4.4 (2.2, 9)	0.01^*∗*^	0.00^*∗∗*^	0.00^*∗∗*^	0.00^*∗∗*^	0.00^*∗∗*^

^a^ANOVA results of the agents in SW, SG, and DG. ^*∗∗*^ *P* < 0.01, ^*∗*^ *P* < 0.05.

^b^Carcinogenic PAHs.

**Table 4:** Comparison of PAHs between risk and control groups by water source.

	Risk group GM (95% CI) (ng/L)				Control group GM (95% CI) (ng/L)				*P* (risk versus control)			
	Surface water (SW)	Shallow groundwater (SG)	Deep groundwater (DG)	*P* ^a^	Surface water (SW)	Shallow groundwater (SG)	Deep groundwater (DG)	*P* ^a^	*P* (SW)	*P* (SG)	*P* (DG)	*P* (all)
Nap	561.0 (294.7, 1068.0)	181.7 (97.9, 337.5)	161.8 (94.1, 278.2)	0.00^*∗∗*^	984.0 (172.1, 5625.6)	139.0 (35.9, 538.1)	230.5 (52.6, 1010.9)	0.12	0.41	0.70	0.66	0.86
Acy	17.9 (12.0, 26.8)	10.1 (7.3, 14.0)	8.2 (5.9, 11.3)	0.00^*∗∗*^	43.2 (16.7, 111.7)	6.7 (2.9, 15.6)	9.3 (4.1, 21.5)	0.01^*∗*^	0.04^*∗*^	0.17	0.75	0.89
Ace	28.6 (16.3, 50.1)	10.9 (6.3, 19.2)	10.5 (6.7, 16.5)	0.01^*∗*^	46.2 (9.9, 215.0)	6.5 (2.1, 20.5)	8.8 (2.4, 32.5)	0.07	0.48	0.40	0.74	0.62
Fl	144.4 (84.8, 246.1)	57.2 (34.6, 94.6)	53.9 (34.2, 84.7)	0.01^*∗*^	278.6 (60.9, 1274.6)	42.8 (13.8, 132.1)	48.4 (13.3, 176.3)	0.07	0.30	0.58	0.65	0.73
Phe	206.4 (117.1 363.7)	78.8 (44.1, 140.7)	88.5 (54.0, 145.0)	0.03^*∗*^	475.5 (90.6, 2494.7)	94.0 (27.4, 323.0)	82.4 (22.1, 308.0)	0.12	0.23	0.78	0.90	0.57
Ant	15.7 (9.3, 26.6)	7.7 (4.6, 12.7)	8.4 (5.1, 13.7)	0.07	37.1 (8.4, 163.1)	12.1 (3.9, 37.3)	7.4 (2.2, 24.9)	0.16	0.19	0.38	0.71	0.43
Flu	46.3 (31.9, 67.0)	32.6 (22.4, 47.2)	21.4 (14.1, 32.4)	0.02^*∗*^	89.4 (32.9, 242.0)	35.6 (12.7, 99.9)	25.1 (10.9, 57.9)	0.14	0.15	0.84	0.72	0.37
Pyr	35.1 (25.3, 48.5)	32.0 (22.5, 45.6)	24.3 (18.4, 32.1)	0.26	67.7 (30.1, 152.1)	27.4 (13.1, 57.6)	20.8 (9.9, 43.6)	0.08	0.09	0.71	0.54	0.93
BaA	4.6 (3.2, 6.6)	3.9 (2.6, 6.0)	3.2 (2.3, 4.4)	0.38	6.4 (3.5, 11.5)	4.5 (1.7, 11.7)	3.2 (1.7, 6.1)	0.41	0.44	0.80	0.97	0.64
Chy	16.7 (12.2, 22.9)	10.7 (7.5, 15.2)	8.9 (6.8, 11.8)	0.02^*∗*^	28.9 (12.5, 66.5)	11.9 (4.8, 29.6)	9.3 (5.0, 17.0)	0.10	0.16	0.79	0.92	0.44
BbF	3.5 (2.5, 5.0)	2.9 (2.1, 4.2)	2.3 (1.6, 3.3)	0.24	2.6 (1.2, 5.9)	2.7 (1.2, 6.2)	2.9 (1.7, 5.1)	0.97	0.47	0.82	0.54	0.83
BkF	26.4 (15.1, 46.0)	12.9 (7.9, 21.2)	17.7 (10.9, 28.8)	0.37	33.7 (17.2, 66.2)	9.1 (2.5, 32.7)	13.2 (4.5, 38.6)	0.31	0.73	0.70	0.54	0.64
BaP	9.6 (7.1, 12.9)	7.3 (4.8, 11.2)	4.2 (3.1, 5.7)	0.00^*∗∗*^	7.7 (3.6, 16.2)	4.5 (2.8, 7.4)	4.5 (2.6, 7.9)	0.28	0.52	0.25	0.85	0.35
IcdP	8.2 (4.9, 13.7)	7.1 (4.7, 10.7)	5.2 (3.6, 7.3)	0.43	12.7 (5.4, 29.6)	8.1 (5.3, 12.2)	5.3 (1.9, 14.9)	0.50	0.69	0.61	0.65	0.92
BghiP	8.8 (6.2, 12.7)	6.0 (4.4, 8.3)	4.7 (3.5, 6.3)	0.02^*∗*^	13.5 (5.9, 30.7)	3.9 (1.7, 8.9)	5.7 (2.9, 11.4)	0.04^*∗*^	0.29	0.21	0.54	0.88
∑PAHs	1274.7 (731.3, 2221.7)	555.9 (340.5, 907.4)	475.4 (303.3, 745.3)	0.03^*∗*^	2308 (492, 10834)	494.6 (155.9, 1569.5)	551.1 (159.6, 1903.1)	0.18	0.65	0.69	0.31	0.75
∑(PAHs-c)^b^	65.9 (46.3, 93.9)	38.7 (25.9, 57.8)	35.1 (25.1, 49)	0.03^*∗*^	79.4 (33.4, 188.7)	39.4 (17.3, 90.1)	30.1 (13.8, 65.8)	0.16	0.72	0.97	0.64	0.85
BaPeq	14 (9.2, 21.1)	7.5 (4.4, 12.7)	7.6 (5.4, 10.6)	0.18	16.8 (6.9, 41.1)	6.6 (2.8, 15.9)	6.1 (2.2, 17.1)	0.15	0.90	0.68	0.58	0.55

^a^ANOVA results of PAHs in SW, SG, and DG. ^*∗∗*^ *P* < 0.01, ^*∗*^ *P* < 0.05.

^b^Carcinogenic PAHs.

**Table 5:** Comparison of PAHs between risk and control groups by season.

	*P* value in winter season (risk versus control)			*P* value in summer season (risk versus control)		
	Surface water	Shallow groundwater	Deep groundwater	Surface water	Shallow groundwater	Deep groundwater
Nap	0.53	0.06	0.73	0.84	0.99	0.38
Acy	0.09	0.01^*∗∗*^	0.34	0.20	0.61	0.47
Ace	0.96	0.37	0.14	0.84	0.08	0.80
Fl	0.96	0.33	0.10	0.40	0.46	0.84
Phe	0.96	0.67	0.41	0.28	0.80	0.80
Ant	0.80	0.39	0.42	0.20	0.37	0.55
Flu	0.56	0.88	0.62	0.22	0.61	0.98
Pyr	0.58	0.62	0.80	0.13	0.94	0.37
BaA	0.04^*∗*^	0.46	0.77	0.55	0.46	0.73
Chy	0.21	0.99	0.84	0.37	0.66	0.42
BbF	0.17	0.82	0.69	0.93	0.86	0.24
BkF	0.84	0.88	0.58	0.66	0.40	0.03^*∗*^
BaP	0.79	0.62	0.93	0.31	0.24	0.96
IcdP	0.88	0.70	0.69	0.76	0.90	0.89
BghiP	0.69	0.11	0.46	0.43	0.65	0.81
∑PAHs	0.80	0.46	0.85	0.57	0.96	0.61
∑(PAHs-c)^a^	0.89	0.86	0.67	0.51	0.35	0.39
BaPeq	0.91	0.84	0.68	0.73	0.29	0.73

^a^Carcinogenic PAHs. ^*∗∗*^ *P* < 0.01, ^*∗*^ *P* < 0.05.

**Table 6:** Descriptive statistics for deterministic and probabilistic carcinogenic risk assessment.

	Mean	SE	P5	P50	P95
Deterministic approach					
Total	7.34 × 10^−6^	9.79 × 10^−7^	1.94 × 10^−7^	4.98 × 10^−6^	1.77 × 10^−5^
Control group (*N* = 117)	5.04 × 10^−6^	6.41 × 10^−7^	1.16 × 10^−7^	4.88 × 10^−6^	1.77 × 10^−5^
Risk group (*N* = 115)	7.88 × 10^−6^	1.19 × 10^−6^	1.94 × 10^−7^	5.10 × 10^−6^	2.19 × 10^−5^
Type of water					
Surface water (*N* = 82)	9.09 × 10^−6^	1.68 × 10^−6^	3.90 × 10^−7^	7.32 × 10^−6^	1.87 × 10^−5^
Shallow groundwater (*N* = 90)	8.03 × 10^−6^	2.05 × 10^−6^	8.54 × 10^−8^	5.15 × 10^−6^	2.19 × 10^−5^
Deep groundwater (*N* = 90)	5.19 × 10^−6^	1.22 × 10^−6^	5.90 × 10^−7^	3.22 × 10^−6^	1.20 × 10^−5^
Probabilistic approach (*N* = 10000)					
Total	1.36 × 10^−5^	2.33 × 10^−7^	1.93 × 10^−7^	6.10 × 10^−6^	5.09 × 10^−5^
Control group	9.61 × 10^−6^	1.76 × 10^−7^	1.26 × 10^−10^	6.57 × 10^−6^	2.90 × 10^−5^
Risk group	1.51 × 10^−5^	3.49 × 10^−7^	1.97 × 10^−7^	5.79 × 10^−6^	5.68 × 10^−5^
Type of water					
Surface water	1.63 × 10^−5^	2.19 × 10^−7^	3.72 × 10^−7^	9.34 × 10^−6^	5.43 × 10^−5^
Shallow groundwater	2.11 × 10^−5^	7.00 × 10^−7^	1.17 × 10^−7^	4.60 × 10^−6^	8.84 × 10^−5^
Deep groundwater	9.16 × 10^−6^	1.39 × 10^−7^	2.64 × 10^−7^	5.05 × 10^−6^	3.12 × 10^−5^
